# Supplementary figures and images for: Analysis of miRNAs in Osteogenesis imperfecta Caused by Mutations in COL1A1 and COL1A2: Insights into Molecular Mechanisms and Potential Therapeutic Targets
Source: Pharmaceuticals (Basel). 2023 Oct 4;16(10):1414. doi: 10.3390/ph16101414 (PMC10609877; doi:10.3390/ph16101414)

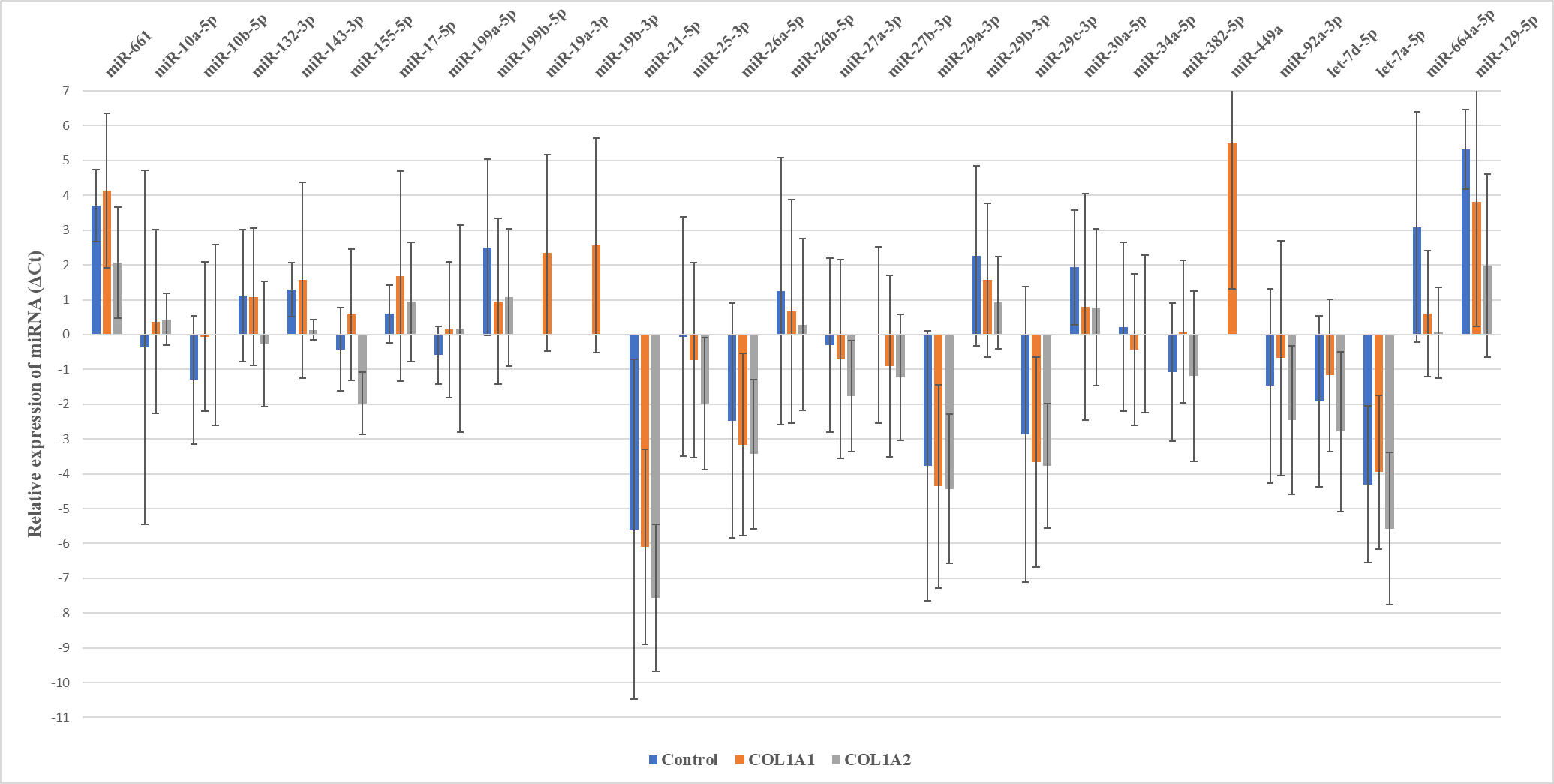

Supplement: Supplementary file 1 [file pharmaceuticals-16-01414-s001.zip › Supplementary Fig 1 The relative expression of the tested miRNAs in subjects with mutations in the COL1A1 and COL1A2 genes and the control cells.tiff]
